# Making Policies with Data: The Legacy of the PoliVisu Project

**DOI:** 10.1007/978-3-030-63693-7_8

**Published:** 2020-11-02

**Authors:** Freya Acar, Lieven Raes, Bart Rosseau, Matteo Satta

**Affiliations:** 1 DASTU, Politecnico di Milano, MILANO, Italy; 2 DASTU, Politecnico di Milano, MILANO, Italy; 3 Digitaal Vlaanderen, Brussels, Belgium; 4 Digitaal Vlaanderen, Brussels, Belgium; 5 Dienst Data En Informatie, Bedrijfsvoering, Stad Gent, Belgium; 6 Digitaal Vlaanderen, Brussels, Belgium; 7 Issy Média, Issy-Les-Moulineaux, France

**Keywords:** Data driven policy making, PoliVisu project

## Abstract

The PoliVisu project has the goal to investigate the potential of data use and visualisation in urban policy making. The project has explored how data supported policy making is adopted by public administrations and what we can learn from their experience. This is done by enrolling pilot cases with different and specific policy problems. From the experience of the PoliVisu pilots the influence and added value of data in the policy making process is assessed. Considering the recent “shake” in data production and use, PoliVisu has adopted four driving questions, as follow: what are the new roles data can play in the policy making process?, What is the added value of data for policy making? How can innovative visualisations contribute to improve the use of data in policy making processes? To what extent can an increased adoption of data affect the policy making process? How is the data shake affecting the involvement of non-institutional actors in the policy making process? This paper explores these questions, by presenting the experiences and the lessons learnt, also focussing on specific pilots’ initiatives and results.

## Data Supported Policy Making Through the Eyes of the PoliVisu Pilots

The digital era and the rise of digital data have opened up possibilities for data supported policy making leaving some questions open, including what is data supported policy making, and what does it imply. “Data supported” is a trendy term that is often used and has a different meaning for every user.

“Data supported policy making” hints at a collaboration, almost a symbiosis, between data and policy making. Nevertheless, the way this collaboration works and to what extent this symbiosis is effective, still need to be investigated (see Charalabidis 2021 for an overview of the main barriers for data supported policy making). The PoliVisu project represents a step forward in this direction. “Policy making” is a process focussing on a policy issue, which is often a complex cluster of problems that encompasses many views and has repercussions on a variety of urban dynamics and domains.

Data management should form the backbone of any process that involves collecting, storing and using city data. PoliVisu’s experience in pilot Cities and Regions has made it possible to identify the fundamental steps needed to deliver an effective data management strategy. These steps involve taking clear and early actions on data literacy, collection and readiness.

Evidence to support PoliVisu’s approach for dealing with data has mainly been collected from five pilots with differing size, data readiness levels and needs, Pilsen (Czech Republic), Issy-les-Moulineaux (France), Ghent, Mechelen and the Flanders Region (Belgium). By exploring different city situations from data, scenario, policy making and administrative points of view, PoliVisu is better able to define a flexible methodology that can be leveraged by a wide range of other cities.

These differences are best illustrated by the example of Issy-les-Moulineaux, which is part of a larger metropolitan area (Île-de-France) and so has to deal with various data sharing and competence issues that are usually not applicable to smaller, stand-alone cities. In the case of Issy-les-Moulineaux, getting the necessary data for its locale requires a very close-knit collaboration with other public bodies and even private companies, e.g. Be-Mobile.

Similarly, for Ghent and Mechelen, the challenge was to obtain the necessary data from third parties as no in-house data was available for the planned use case. This in turn required the city to form partnerships with local service suppliers-cum-data owners. Finally, Pilsen and Flanders Region worked using their own data, but because they are using different sources (Traffic sensors in the first case and ANPR cameras in the second) they have to deal with another issue, i.e. the integration of a large number of data sources.

All the activities of the public authorities have been focused on defining co-creation actions that might make the implementation of data (and related tools) processes more effective.

The experiences of the Polivisu pilots revealed some common challenges that can be synthetized in the following questions:

- what are the new roles data can play in the policy making process? what is the added value of data for policy making?
- how can innovative visualisations contribute to improve the use of data in the policy making process?
- to what extent can an increased adoption of data affect the policy making process?
- how is the data shake affecting the involvement of non-institutional actors in the policy making process?

In the following paragraphs the four questions are discussed in relation to the perspectives adopted by pilots in the experiments and attempts made while searching possible responses.

### Data for Dialogue

Using data as a supportive resource for dialogues has different communication aspects and has various impacts on the target groups. PoliVisu tried to manage two challenges. The first was to close the chasm between diverse policy domains in and between government organisations. The second was using data visualisations as a communication tool for policy making to citizens.

The first challenge was to facilitate the communication and the cooperation between diverse policy domains and government organizations. In the pilot of Issy-les-Moulineaux for example the policy issue was traffic congestion. After thoroughly investigating the problem, it became clear that the congestion was not caused by citizens of Issy, rather by drivers working there or transiting to reach Paris. This required the collaboration with various stakeholders, in the public and private sector, which brought to discuss the problem with key actors who need to be aligned on the problem setting, the language and the available opportunities (see Raineri and Molinari 2021). Thanks to data and data visualisations the policy issue can be observed through clear images and dialogue activated towards a shared understanding and solutions development.

However, understanding data and talking about data-related evidences require a certain level of data literacy. By extracting visualisations from the data, the data becomes more comprehensible and improves effectiveness of the communication.

The Flanders accident map managed to set up cooperation on accident data interpretation between the Federal police and the traffic safety institute. This approach had led to a better-streamlined interpretation of the data and cooperation to locate the accident spots better. An online accident map, visualising road accidents, is an excellent tool to give all involved parties in traffic safety insights in where the most serious accidents happen. Also, citizens have the necessary information to elaborate on the local traffic safety situation and discuss traffic safety in their (local) community. The Flanders accident visualisation can be seen as an enabler for local policy making to improve, for example, the traffic situation around schools.

Pilsen used a traffic model as a successful communication instrument for citizens. Traffic models were long considered as an internal tool of the traffic department to perform what-if analysis. An open visualisation interface allowed citizens to elaborate on the impact of the planned road works on their neighbourhood at different moments. Citizens could find out if their neighbourhood were affected positively or negatively. The user-friendly traffic model visualisation was accompanied with practical information about the road work planning, necessity and expected improvements.

Mechelen has an active policy of rolling out schoolstreets. The city of Mechelen wants to know the effect of the schoolstreet on the traffic in the school street itself and on the surrounding streets. Parents were involved to install a traffic counting device (telraam) behind their window and a public dashboard is able to visualise the life situation and to measure the long-term effect of the schoolstreet. The results were used to dialogue with the school community and the surrounding neighbourhood. As a result the expected negative impact as expected by some people living in the neighbourhood wasn’t the case.

Issy-les-Moulineaux created, in collaboration with a local startup called MyAnatol, a dashboard to analyse traffic data on the main axes of the city. But the analysis of cars alone was not enough in a period, due to COVID-19 outbreak, that saw an increase in measures in favour of bicycles. This usage has been added to it. The innovative side is also linked to the publication in open data of the data from the dashboard and all the traffic measurements, which are therefore now updated weekly and available to everyone on the data.issy.com portal. This data allowed the City to evaluate its policies about bikes introduced after the lockdown, but also to have a wider view on bikes use, with a comparison between 2019 and 2020.

The four examples above show three different kinds of interaction between stakeholders and citizens: the accident data and map are beneficial for co-creative policy design to improve the local traffic situation; the Pilsen road works communication turned out to be a good communication instrument for policy implementation; the dashboards in Issy-les-Moulineaux and Mechelen turned out to be very useful for evaluation purposes.

### Between Precision and Usability

In data supported policy making, data and data visualisations are used to enable discussion between the actors that are involved in the policy making process (Androutsopoulou and Charalabidis 2018). Visualisations, in particular, are essential to facilitate this conversation, especially if supplied through the adoption of tools enabling dynamic visualisations.

Tools allow the gathering of data, the combination of different data sets and different visualisation of data. At different stages of the policy making process different tools can be more or less suitable as each stage may have specific needs (Verstraete et al. 2021) At the early stage of the policy making process, an easy to use tool can be preferred as not requiring special skills or abilities. When more specialized analyses are necessary more specific and powerful tools may be adopted that require data scientists’ expertise for the production of more precise, reliable, and effective results. Finally, when the results are discussed with policy makers, again an easy to use tool may be preferred.

In the Flanders and Pilsen road accident maps QGIS was initially used to test the data suitability; later on a BI (Business Intelligence) tool allowed more precise analysis to gain insights from the relationship between accident data and other, more contextual, data sets; finally QGIS was used again for data editing and for the test of the visualisation layout.

The Ile-de-France Region has launched the platform “Ile de France Smart Services”. The city of Issy-les-Moulineaux have been between one of the first Cities to have signed this agreement. The implementation of these services is the result of an unprecedented partnership approach around data between public and private actors, as it represents also a common data portal including datasets and data visualisation tools.

Using tools in coherence with the policy making specific needs and sharing their use among different offices of the same organization or with other organizations gives different advantages. First if more local governments adopt the same tool this can significantly reduce the public cost of tool development or acquisition. Second, it also allows for more straightforward communication between public administrations because the tool and visualisations are the same. Also some pre-processed data can be reused since they are already digested by the tool. However, it should always be possible to change and improve the tools in coherence with policy making needs: flexibility and possibility to experiment should be the main characteristics of tool fit for data driven policy making.

PoliVisu developed the open WebGLayer tool for advanced visualisation tool and the open Traffic Modeller tool for traffic predictions. Both tools allowed Flanders and Pilsen to create tailored visualisations of their traffic safety and accident map and allowed them to open traffic models to the public. The rather limited differences in the data content, made it possible to deploy without much effort tailored visualisations.

Then, from tools and analyses, visualisations arise. Visualisations are an effective way of communication with the variety of people engaged in the policy making process. Visualisations can be used to start a conversation. They can show the situation as it is, where difficulties are situated, and how policy decisions can change and improve the situation. Visualisations are a work in progress. They can continuously be improved to ensure that they are easy to understand and effective in reflecting the policy problem with reduced bias. Constructing a useful visualisation is a challenging and time-consuming task. The use of suitable visualisations responding to specific discussion or dialogues requirements asks for the capacity to balance between precision and usability of the visualisations s as well as for a certain flexibility in the adoption of the best tool.

### Proneness to Iterative Process

To move from policy making to data supported policy making asks for data maturity and data literacy in the organization.

As already highlighted by Verstraete et al. (2021) the policy making is structured on to three stages: policy design, policy implementation and policy evaluation. In the policy design stage, the context of the policy problem is assessed, a policy formulation is constructed, different scenarios are hypothesized and analysed and subsequently a policy decision is made. In the following stage the policy decision is implemented, through an implementation plan. The effects of the policy implementation are monitored and apt communication concerning the policy is vital in this stage. In the final stage, when the policy decision has been executed for some time, the policy evaluation takes place through an impact assessment. Based on this assessment a restructuring of the problem might take place, resulting in a new policy problem.

This policy making model is already iterative by nature. After a policy decision has been evaluated it often results in a new policy problem. Furthermore, at any stage in the policy making process it is possible to go back a few steps and restart the process guided by the lessons learnt. This is the key core of the impact of data on policy making: it is transformed into a trial/error process where it is essential to register, reuse and share the outcomes and learnings developed throughout the process (Concilio and Pucci 2021). By virtue of these learnings the data maturity of the organization, the public administration, grows, bringing new insights to the field. These insights in their turn can indicate that it might be opportune to go back to a previous step in the policy cycle. The bigger availability of data augments the option to check the policy measures during the implementation so reducing the risk for irreversible mistakes.

In Pilsen and Mechelen, thanks to the availability of a traffic model and a list of the planned road works, the impact of multiple planned road works and deviations can be simulated. In Mechelen, a service was tested to simulate the immediate effect of the occupation of the public domain as part of the approval procedure, including signalling plan and electronic payments.

Furthermore, depending on the step and stage of the policy cycle different issues arise (Verstraete et al. 2021 for a complete overview of the types of data analysis in each step of the process). In the first stage, the policy design stage, the policy and data issues are more related to the scope of the policy problem. By setting up a context and through the analysis of scenarios data has a guiding role in this stage, allowing more insight in the context of the policy problem. In the policy implementation stage the questions are more related to monitoring and fine-tuning. In this stage data is used to follow up on the policy decision and adapt or adjust the policy decision if necessary. In the final stage data use is more related to analyses and checks, whether the policy decision was successful, sufficient, and how it affected the domain of the policy problem and other, related domains.

Additionally, data has the property of moving quicker through the policy cycle than the policy itself. In consequence the results and learnings from the data can have an influence on the policy making process at any given point and require flexibility and an agile performance from the actors involved in the policy making process.

### Actors Involved in Data Supported Policy Making

Data supported policy making is a complex process that involves many different actors. A network analysis of the PoliVisu pilots showed that at least 20 actors were involved in every pilot (see Lanza 2021). This includes partners both internal and external to the organization. For every pilot a core group could be distinguished from peripheral partners. It is clear that the core group is a multidisciplinary team where people with varying expertise work closely together.

Agile and effective data-supported policy making is an interdisciplinary challenge and requires the combination of multiple perspectives (Walravens et al. 2021). At least a policy perspective and a data perspective have been identified. From the data perspective different competences are required. These different competences are hardly found in one multi-talented person. Moreover, one person will never have the time to deal with the multitude of tasks related to data-supported policy making. On the other hand, most organisations will not be able to install a complete multidisciplinary team at once. A good starting point is to focus on three general profiles: a data-engineer to cover data management and development needs a general data-analyst to cover the data-analyst and data science needs; and a policy (decision) maker. Gradually, with the evolution to more complex analyses and a more mature data-supported policy making, the organisation can invest in specialized roles such as expert data-analysts, statistician, data scientists and researchers.

In the Ghent pilot several (internal and external) partners are at work to handle the policy problem. A data-engineer is at work to maintain the datasets and provide a framework to work with the datasets. Data-analysts are working within the public administration at the office of data and information and externally at the telecom provider. Finally, a close collaboration exists between the office for data and information and the policy makers involved in the student housing problem setting.

In Issy-les-Moulineaux, the City created a dashboard and related KPIs to connect the various departments with policy makers and, at the same time, providing a simplified version for citizens to use data to help them to have better information about the impact of policies.

A data-driven organisation must ensure through its organisational structure, the collaboration of the different actors in the policy making process. Related to the data activities, three main organisational forms can be distinguished: a centralized organisational structure, a decentralized structure and a balanced hybrid form between these two.

In a decentralized organisation, business or policy domain units develop their own data analytics teams. This promotes the responsiveness of the dedicated data teams to the priorities of the units. Also, since the data teams are embedded in a policy domain, they are likely to develop thorough domain knowledge. However, isolated decentralized teams might suffer from siloed data expertise, the inability to develop an organisational data strategy and the lack of broader managerial focus. Smaller decentralized teams probably will not have dedicated data engineers and developers. For ad hoc analyses this might not be a great concern, but the analysts will be unable to deploy relevant results to productional data-pipelines and automated analyses. Data analysts might also struggle with flexible data access and the deployment of generic analysis and visualisation tools.

A centralized structure has many advantages in terms of talent and knowledge management, the potential to develop a cross-departmental data strategy and a broader managerial focus. Still, a central unit might face important challenges concerning the allocation of sufficient resources to individual business units and flexible responses to domain priorities. Centralized data teams need to invest extra time to gather sufficient domain knowledge. The installation of a centralized data team can be a good starting point to engage in data-supported policy making. Sooner or later, organisations will feel the need to evolve to a more hybrid form to balance the advantages and challenges of both the centralized and decentralized organisation structure.

Because of the specific knowledge required by working with (big) data for policy making it happens that the data experts working for the data providers become effective collaborators of the policy making organization so transforming it into an hybrid structure.

The city of Ghent started working with the data scientists from Proximus to ensure a reliable data analysis. This collaboration showed to be a win-win process for both parties. The city learned how to work with this kind of big data sources and Proximus learned how a city operates and how it formulates its requirements to (big) data.

Issy-les-Moulineaux collaboration with a local startup, My Anatol, and its data specialists. This made possible for the City to access data and skills and the startup to improve its offer for public authorities, being able to have a real proof of concept in real, through the various requirements and feedback received from the City.

Actors involved in the policy making process are different: policy makers, operative sectors of the public administration, an office for data and information within the public administration, technical service providers, data providers and the public. Every actor has a specific task or purpose. It is highly relevant to identify which policy makers you need support from, which operative sectors of the public administration you require information and who in the office for data and information that can aid with the data management plan and find the link between data and the policy issue. External to the organization, other actors might be necessary either because they are data owners or because they can help with data analysis and visualisation. The pilots realized that identifying and interacting with diversified actors since an early stage of the policy making process augment the productivity of the process.

In conclusion, for data driven policy making, collaboration between many different actors, internal and external to the organization, is required. Because of the complexity of the process, it is advisable to set up structures and come to agreements at the start of the process.

## Bottlenecks and New Practices Detected in Policy Making

The project showed that the way to the full exploitation of data potentials in policy making is still long. PoliVisu pilots experienced several bottlenecks in data availability and use that still represent important limitations to effective processes of data driven policy making. Such bottlenecks, managed by the project pilots, highlighted important adaptation that gave rise to new use of data.

The findings mentioned in this section have been collected through the observation on various pilots mentioned in the first section of this paper. This work made the actual development, implementation and monitoring of local policies in four cycles, feeding the results back into the overall project’s solutions.

### Bottlenecks

The different pilots of PoliVisu started the project with a high degree of knowledge about open data and work with simple datasets. At the same time, they had ambition, but a limited knowledge on use of big data and smart visualisations.

Those pilots entered then into the project with some interesting scenarios, but those were created without a real certainty about the possibility to really deploy them successfully.

One of the questions that PoliVisu wanted to answer with the support of practical cases, at least for local public authorities, is “Do we really have a resistance to innovation processes in the public local authorities?”. According Ritchie (2014) the theories claiming an anti-innovation approach of governments have somehow a base of truth, but he also considers that it is still needed to have a deeper research to understand the reasons.

Table 8.1 mentioned bottlenecks, detected all along PoliVisu, seem to show how the resistance is somehow structural and often not just related to individuals.

**Table 8.1:** Bottlenecks faced by the PoliVisu project

Data literacy	According to the Data Literacy project, “76% of key business decision-makers aren’t confident in their ability to read, work with, analyse and argue with data”. The PoliVisu project investigated the issue of data literacy using its own survey which made confirmed this issue. At the same time, this was underlined by most of the pilots (all, but Ghent), at the beginning of the project, which declared not to have data scientists or analysts in their teams
Data ownership	All pilots had to deal with data ownership, but in two cases this is particularly true. Ghent and Issy-les-Moulineaux found themselves in a situation in which they had the obligation to get the data from private bodies, consequently, to have a real work to benchmark the market and to find affordable solutions. This is extremely time consuming and it plays a, potentially negative, role in deployment of data projects
Data fragmentation	Often the various municipalities and/or other public bodies do not share data or do not have compatible formats and policies. This is a real point as it makes data often non exploitable and it was experiences by all the pilots of the project
Fragmentation of jurisdiction	The fragmented data is also related to the division of competences between various bodies. This was particularly impacting in Flanders and Issy-les-Moulineaux due to their geographical configuration as Flanders has to deal with various Cities and Issy-les-Moulineaux is part of a big urban agglomeration which is very dense, and it has many decision levels
Data privacy	Data privacy policies oblige, particularly with the new GDPR regulation, to be extremely careful to any potential breach of data privacy. In particular, the case of Ghent in which mobile data was used is key. Even if the data was anonymized, the detection of a person can represent a real issue, consequently important steps need to be taken with, next to anonymization, aggregation of data

The bottlenecks listed above are all obviously important, but there are some having somehow a heavier impact.

In particular, the PoliVisu experience in its Cities and public authorities shows that private companies often hold the most useful datasets, as often public data (and competences), is fragmented. This evidence shows an unbalanced relation between the private and public sector which may explain, at least partially, a reluctance to innovate in data in many cities and public authorities.

### New Practices and Knowledge

As it is now clear to everyone, an increasing reduction of specific budgets of Cities (including the shift of priorities), and public authorities in general, is today a reality. This reduction cannot be tracked with a clear trend, being highly different country by country, but it follows the trend of national public budgets. The Council of Europe, already in 2011, was warning about potential negative effects, even recognising the need of contribution of public authorities, in an ad hoc publication.

As explained in the previous paragraph, the PoliVisu experience and the ones of its Cities and Regions show how a data transition requests high level investments, not without risks, for the public authorities both from a data and visualisations point of view. One of the major findings of PoliVisu, as reported above, has been the detection of an unbalanced relation between the private and public bodies, being often the most valuable data held by private companies.

In this framework, PoliVisu pilots showed how it is possible to move through this bottleneck and the ones mentioned above, partially or totally, adopting some new practices, summarized in Table 8.2.

**Table 8.2:** Emerged practices

Awareness of data value	The various pilots were already aware of the value of data in itself, having already advanced open data strategies in place and a vision related to data. At the same time, in particular at decision makers level, this knowledge was just guessed. During the project, the meetings with policy makers have totally unlocked the potential of pilots that could start working 100% on such projects with a snowball effect. The various pilots that came out or could be deployed anyway in Issy, Mechelen and Pilsen in COVID-19 time are really an example of how pilots took data as a real resource
Use of dashboards	The pilots at the beginning of the project had quite interesting ideas about tools and solutions, but those were theoretical and not often realistic. As the project moved on, pilots had to deal with reality, and they could finally find solutions. Every pilot has its own specifications, but a common point was the use of dashboards to have analyses of data. Those tools are useful to connect the policy makers with their operational departments and, in some cases, directly to citizens to make better accepted and/or understood some policies
Co-creation projects	The PoliVisu’s cities have started the project with scenarios that looked “self-standing”, but the various bottlenecks met and PoliVisu itself pushed them to involve more and more other stakeholders. Finally, all pilots had constructed co-creation projects in which other private and public partners played a role. This was extremely evident in Ghent, Issy and Flanders, but absolutely true also in Pilsen where applications created by local start-ups, universities and associations were mixed with the City open data portal to improve the information

Those practices, collected through observation and feedback received, made possible to highlight how the process in adoption of data in policy making has an upward trend as Cities and public authorities may have a slow start, but, as soon as they start, they will be improving quicker and quicker with time. The improvement will also ameliorate the communication to citizens, making also possible to unlock more and more funds, which will support the whole process.

To do so, it is really important to engage the City in co-creation processes and projects which make easier to unlock this potential, giving access to the City to data and skills that would, otherwise, never be available.

## Conclusions

Through its pilots and their stories, PoliVisu has showed the potentials of Open and Big Data in policy making. This closing section aims at wrapping up the whole stories of the project, giving also some recommendations to deploy, in cities, projects through the use of data.

### Lessons Learnt from the PoliVisu Project

In data supported policy making, data is used to commence a dialogue. Data can activate dialogues, public dialogues, concerning a policy problem, and can support arguments and visions concerning the challenge at hand. Adding data and data visualisation to the conversation allows for a better understanding of the problem, the context, and the possible effect of policy decisions. However, data can support dialogues about policy issues without neglecting the complexity of the policy process where many viewpoints and approaches are intertwined. Vision, knowledge and experience still represent the fundament of policy making, and data can contribute to a part of that knowledge.

Getting knowledge from data is a challenging process. Visualisations aid everyone involved in the dialogue to understand the data. We distinguish between three types of visualisations based on the stage of the policy making process in which they are used. At the beginning, when only an exploratory analysis is performed and the dialogue concerns preliminary results, visualisations are used that are accessible and easy to use. The goal is to transfer, explore and discuss intermediate analysis results. This allows for the exploration of some general trends, but no in-depth analysis and results can be displayed.

Then, when the results are analysed more in depth, flexible and powerful visualisation tools may be more appropriate. These visualisations require expertise from data scientists. The visualisations allow the data scientist to better understand the data and obtain results that would not be visible with more general visualisations.

Once the in-depth analyses have been performed, visualisations are developed that allow policy makers and a broader audience to query the data. Easy to use visualisations support policy makers when monitoring.

All these visualisations types should be flexible and evaluated at certain points in time. New issues might arise, and new information might come to light. It is important to note that it is not necessary to stick with a visualisations type once one has been chosen. It is always possible to change the visualisations that need to be flexible to best support the policy making process.

The Polivisu project also showed how data supported policy making is an iterative process. At every stage of the policy making process it is possible to go back and start again by considering the lessons learnt. Likewise, the data activities related to data supported policy making happen in an iterative manner as well. As shown in the policy-oriented data activities framework (as described in Verstraete et al. 2021) there is a continuous process where analysis results go back and forth between the policy maker and the data scientist with the ultimate goal to obtain sensible results that can be used to support policy making through close collaboration between the policy maker and the data scientist.

Data supported policy making is a team effort that requires specific and varying expertise from the people involved. In short, data supported policy making is a complex and challenging process that requires communication and collaboration between a diversified group of actors. These actors, and the organization, will gain data maturity through time. This might come across as a slow process, but every small change has a significant value.

### Some Recommendations

The PoliVisu project highlighted some pathways for the situation to be improved towards a more effective integration of data in policy making. They are all illustrated in the following final Table 8.3.

**Table 8.3:** Recommendations from the PoliVisu project

Increasing Data Literacy	As reported above, Data Literacy is a real blocking point to deploy a good strategy. To this end, it is necessary to hire and to engage data analysts and scientists, at least as subcontractors. This is a key point to be successful
Breaking Silos	Large and medium sized municipalities, in fact, are normally “siloed” structures, often not well connected between them. In this sense, even the data produced or stored by these silos are considered as a sort of exclusive property, which is not shared with other silos, even if that may bring benefit to the Municipality as a whole. Siloed organizational status is reproduced in data production and management. The best way to tackle this barrier is to create cross-cutting working tables with various services, starting from the ones that are considered more adapted and ready for a full digital transformation
Showing data value to key internal players	The second relevant element of the political culture affecting the management of data is related to the role played by data in the Municipality procedures. Data is rarely, almost never, considered as a useful resource per se; it is rather seen as a functional component of bureaucratic procedures and, as such, not considered as a relevant output of any process. This reduces the attention to data production and management and does not include any scenario of data re-use or utilization in other activities or processes. It is clear that failure in considering data as public good finds its origin in the (merely) bureaucratic approach to public service production and supply; one could even say that public services themselves are not considered or managed as common goods. To move through this barrier, it is needed to show the value of data, creating some first useful applications in a pilot mode, the positive reactions of citizens and external stakeholders will represent a real motivation for City teams
Giving a strong political support to digital transformation teams	The third element is strictly related to individual behaviours, being a project to implement data a real change of paradigm that requests a strong effort in the short term. It is not obvious to have teams of the various departments to “hide” their non-effort to make the internal procedures improved through data. This psychological effect is actually related to the same reasons related to the behaviour shift in mobility, while a person that for 20 years has used the car to go to work, even when not motivated enough, refuses to change, even if confronted with clear proofs that a switch would give him/her an advantage in the medium-long term. The PoliVisu’s Cities experiences showed how a strong political will is absolutely necessary to go through this resistance
Building partnerships with the private sector	PoliVisu has noticed how the construction of private-public partnerships, also with some minor financial contributions of Cities and other public authorities, can drive to the construction of efficient collaborations with good advantages for all parties. Actually, the project could stimulate Cities to look for private partners providing data (Issy) or tools (Pilsen) or both of them (Ghent) and to settle an on the ground collaboration. While this collaboration starts, the project has noticed how Cities start a quick innovation process, showing how the usual anti-innovative approach can change, and private companies show an unusual capacity to support them, also providing, in some cases, investment (Issy-les-Moulineaux and Ghent particularly)
Including data clauses on public procurements	Cities need to show a good capacity to learn from their past mistakes. In particular, the lack of inclusion of clauses in public procurement contracts is one of the biggest lessons learnt from Cities, making those clauses, from now on, fundamental in all public tenders. It is then absolutely necessary to include clauses on public procurements to access the data and, when necessary, to have included also a good format making it quickly usable. Cities should also consider whether, in addition to getting access to the data themselves, the contracts should require the supplier to make the data available as open data or to other private sector actors on a fair and equitable basis so that innovation and societal benefit can be maximised
